# Predicting biliary complications after liver transplantation: a machine learning and nomogram-based exploratory study

**DOI:** 10.1186/s12876-026-04924-0

**Published:** 2026-05-12

**Authors:** Han Zhang, Jinxi Chen, Minghang Zhang, Kezhen Zong, Shanshan Li, Zuotian Huang, Zhongjun Wu

**Affiliations:** 1 Liver Transplantation Center, The First Affiliated Hospital of Chongqing Medical University, No. 1 Youyi Road, Yuzhong District, Chongqing, 400016 China; 2 Department of Hepatobiliary Pancreatic Tumor Center, Chongqing University Cancer Hospital, No. 181, Hanyu Road, Shapingba District, Chongqing, 400030 China

**Keywords:** Biliary complications, LightGBM, Liver transplantation, Machine learning, Nomogram, Risk prediction

## Abstract

**Background:**

Accurate risk stratification of biliary complications (BCs) after liver transplantation (LT) remains challenging. This study aimed to develop and validate a machine learning (ML) and nomogram framework comprising a ML-based web calculator and a clinically interpretable nomogram for post-LT BCs.

**Methods:**

This retrospective study analyzed 133 LT patients (2011–2025), randomly split into training (*n* = 94) and validation (*n* = 39) sets. Predictors were identified using Least Absolute Shrinkage and Selection Operator (LASSO) regression. Eight ML algorithms were trained with 5-fold cross-validation. Performance was evaluated using the area under the receiver operating characteristic curve (AUC), Brier score, and decision curve analysis (DCA). Additionally, SHapley Additive exPlanations (SHAP) were employed to visualize feature importance.

**Results:**

The cumulative incidence of BCs was 38.3%. Four independent predictors were identified: hepatocellular carcinoma (HCC) emerged as a protective factor, while intraoperative crystalloid infusion, preoperative partial hepatectomy, and antiviral therapy were risk factors. Light Gradient Boosting Machine (LightGBM) yielded the highest discrimination in the validation set (AUC 0.753), outperforming standard logistic regression (AUC 0.701). The LightGBM model exhibited satisfactory calibration (Brier score = 0.212) and clinical net benefit. The nomogram provided a static scoring tool, whereas the LightGBM-based web calculator offered precise individual risk estimation.

**Conclusions:**

As an exploratory, proof-of-concept framework and an internal validation effort, the LightGBM-based web calculator offers exploratory but promising predictive accuracy for post-LT BCs, while the nomogram facilitates bedside decision-making through visual interpretability. This combined framework supports a transition from reactive management to proactive, risk-stratified surveillance.

**Supplementary Information:**

The online version contains supplementary material available at 10.1186/s12876-026-04924-0.

## Background

Liver transplantation (LT) remains the definitive curative treatment for end-stage liver disease and selected hepatic malignancies [1, 2]. Despite substantial improvements in perioperative management and survival outcomes, biliary complications (BCs) persist as the “Achilles' heel” of LT, with a reported incidence ranging from 10% to 30% or even higher in complex cases [3, 4]. Encompassing bile leakage, anastomotic strictures, and non-anastomotic strictures, these complications are significantly associated with graft dysfunction, re-transplantation, and increased mortality [5]. Consequently, an effective early risk prediction strategy is critical for guiding postoperative surveillance and improving long-term prognosis [6, 7].

Traditional scoring systems, such as the Model for End-Stage Liver Disease (MELD) and Child–Pugh classification, were originally designed to assess liver disease severity rather than postoperative complications [8]. While previous studies have attempted to develop specific risk models for post-LT BCs, they predominantly rely on conventional logistic regression methods or limited clinical variables, often yielding suboptimal predictive accuracy [9]. Recently, advanced machine learning (ML) algorithms have been explored to capture complex non-linear interactions in transplant data [10]. However, most of these high-performing models—even those published as recently as 2025—exist primarily as complex algorithms without accessible user interfaces (e.g., web calculators), limiting their practical utility at the bedside [11, 12]. Moreover, the inherent opacity of complex algorithms often impedes clinical trust compared to transparent traditional scoring systems [13, 14].

To bridge the gap between algorithmic precision and clinical usability, this study aimed to develop and validate a prediction model for BCs following LT using a novel machine learning and nomogram framework. We integrated a high-performance Light Gradient Boosting Machine (LightGBM)-based web calculator for precise individual risk estimation with a conventional nomogram for bedside visual interpretation. This strategy balances advanced algorithmic accuracy with clinical applicability, providing clinicians with a practical tool for individualized perioperative management.

## Methods

### Study design and participants

This single-center retrospective cohort study was conducted at the Liver Transplantation Center of The First Affiliated Hospital of Chongqing Medical University. We consecutively enrolled patients who underwent liver transplantation (LT) between November 2011 and November 2025. The study protocol adhered to the Declaration of Helsinki [15] and was approved by the Institutional Ethics Committee (Approval No. 2025-845-01). Reporting of this study follows the TRIPOD + AI 2024 statement (Transparent Reporting of a multivariable prediction model for Individual Prognosis Or Diagnosis – Artificial Intelligence) [16].

Inclusion criteria were: (1) adults (aged ≥ 18 years) undergoing primary orthotopic LT; (2) use of grafts from either deceased or living donors; and (3) availability of complete perioperative clinical data. Exclusion criteria were: (1) multiorgan transplantation (e.g., liver-kidney); (2) re-transplantation; (3) pre-existing active infection or severe sepsis; and (4) a follow-up duration of less than 60 days due to early mortality or loss to follow-up. This minimum follow-up period was selected to ensure adequate observation time for early biliary complications and to mitigate competing risk bias associated with early postoperative mortality.

Perioperative management followed a standardized protocol. Surgical procedures were primarily performed using the classic or piggyback orthotopic technique. Living-donor liver transplantation (LDLT) or laparoscopic-assisted procedures were employed in selected cases. Postoperative immunosuppression typically consisted of a calcineurin inhibitor-based regimen combined with mycophenolate mofetil and corticosteroids, adjusted according to the center's standard guidelines [17, 18].

### Data collection and variable definitions

Data were extracted from the electronic medical record (EMR) system by two independent investigators. Candidate variables covered recipient demographics, underlying liver disease, and intraoperative parameters (detailed in Table 1). Predictors were extracted from medical records dated prior to the diagnosis of complications, ensuring assessment was blinded to the outcome. To ensure consistency and reproducibility, specific clinical definitions were applied:

- *Laboratory Variables*: The most recent values obtained within 24 hours prior to surgery were analyzed.
- *MELD Score*: Calculated using the standard formula: 3.78 × ln(bilirubin) + 11.2 × ln(international normalized ratio [INR]) + 9.57 × ln(creatinine) + 6.43 (with bilirubin and creatinine converted to mg/dL) [8].
- *Portal Hypertension*: Defined by the presence of esophageal varices on endoscopy or imaging evidence of collateral circulation [19].
- *Splenomegaly*: Defined as radiological evidence of spleen enlargement (major axis > 12 cm or thickness > 4 cm) on preoperative computed tomography (CT) or magnetic resonance imaging (MRI).
- *Ascites Severity*: Graded based on intraoperative volume quantification: None (0 mL), Mild (< 200 mL), Moderate (200–1500 mL), and Severe (> 1500 mL), in accordance with the severity classification framework [19].
- *Intraoperative Variables*: Fluid balance (e.g., crystalloids, blood loss) and operative duration were recorded by the anesthesia team.

**Table 1:** Baseline characteristics

Characteristic	Total (*n* = 133)	Training Cohort (*n* = 94)	Validation Cohort (*n* = 39)	*P* value
Biliary Complications				0.569
No	82 (61.7)	56 (59.6)	26 (66.7)	
Yes	51 (38.3)	38 (40.4)	13 (33.3)	
Gender				0.704
Male	110 (82.7)	79 (84.0)	31 (79.5)	
Female	23 (17.3)	15 (16.0)	8 (20.5)	
Age (years)	48.37 (11.19)	48.64 (10.64)	47.72 (12.55)	0.668
Hepatitis B Virus				0.646
No	29 (21.8)	19 (20.2)	10 (25.6)	
Yes	104 (78.2)	75 (79.8)	29 (74.4)	
BMI (kg/m²)	22.89 [21.01, 25.39]	23.02 [21.02, 25.48]	21.97 [20.66, 24.88]	0.422
Hypertension				0.791
No	114 (85.7)	81 (86.2)	33 (84.6)	
Yes	19 (14.3)	13 (13.8)	6 (15.4)	
Diabetes				1.000
No	110 (82.7)	78 (83.0)	32 (82.1)	
Yes	23 (17.3)	16 (17.0)	7 (17.9)	
Partial Hepatectomy				0.454
No	106 (79.7)	77 (81.9)	29 (74.4)	
Yes	27 (20.3)	17 (18.1)	10 (25.6)	
Antiviral Therapy				0.831
No	51 (38.3)	35 (37.2)	16 (41.0)	
Yes	82 (61.7)	59 (62.8)	23 (59.0)	
Smoking History				1.000
No	81 (60.9)	57 (60.6)	24 (61.5)	
Yes	52 (39.1)	37 (39.4)	15 (38.5)	
Alcohol History				0.610
No	86 (64.7)	59 (62.8)	27 (69.2)	
Yes	47 (35.3)	35 (37.2)	12 (30.8)	
Hepatocellular Carcinoma				0.845
No	58 (43.6)	42 (44.7)	16 (41.0)	
Yes	75 (56.4)	52 (55.3)	23 (59.0)	
Cirrhosis				0.812
No	6 (4.5)	5 (5.3)	1 (2.6)	
Yes	127 (95.5)	89 (94.7)	38 (97.4)	
Liver Failure				0.740
No	105 (78.9)	73 (77.7)	32 (82.1)	
Yes	28 (21.1)	21 (22.3)	7 (17.9)	
Portal Hypertension				0.834
No	36 (27.1)	25 (26.6)	11 (28.2)	
Yes	97 (72.9)	69 (73.4)	28 (71.8)	
Splenomegaly				1.000
No	32 (24.1)	23 (24.5)	9 (23.1)	
Yes	101 (75.9)	71 (75.5)	30 (76.9)	
Ascites Severity				0.792
None	32 (24.1)	21 (22.3)	11 (28.2)	
Mild	58 (43.6)	41 (43.6)	17 (43.6)	
Moderate	20 (15.0)	14 (14.9)	6 (15.4)	
Severe	23 (17.3)	18 (19.1)	5 (12.8)	
WBC (×10⁹/L)	4.17 [2.79, 5.61]	4.17 [2.75, 6.15]	4.29 [3.28, 5.16]	0.809
Hb (g/L)	116.20 (28.55)	115.59 (28.60)	117.67 (28.72)	0.703
PLT (×10⁹/L)	75.00 [46.00, 113.00]	69.50 [44.25, 111.75]	79.00 [51.50, 117.00]	0.324
ALT (U/L)	41.00 [30.00, 57.00]	41.00 [31.00, 56.25]	41.00 [29.00, 55.50]	0.851
AST (U/L)	48.00 [34.00, 74.00]	48.50 [34.00, 72.50]	46.00 [33.50, 74.50]	0.772
Albumin (g/L)	37.00 [33.00, 41.00]	37.00 [33.00, 41.00]	38.00 [33.00, 41.00]	0.812
Total Bilirubin (µmol/L)	30.40 [17.50, 70.30]	29.75 [17.80, 68.12]	31.00 [17.75, 70.60]	0.658
ALP (U/L)	121.00 [85.00, 196.00]	123.00 [85.00, 184.75]	121.00 [91.00, 217.00]	0.816
GGT (U/L)	55.00 [35.00, 116.00]	56.50 [37.00, 121.00]	48.00 [32.50, 89.50]	0.489
Urea (mmol/L)	5.10 [4.20, 6.00]	5.10 [4.40, 5.97]	4.90 [3.80, 5.90]	0.379
Creatinine (µmol/L)	66.00 [54.00, 78.00]	66.00 [55.00, 78.75]	66.00 [53.00, 75.00]	0.713
INR	1.30 [1.13, 1.65]	1.30 [1.14, 1.65]	1.26 [1.10, 1.61]	0.429
MELD Score	11.65 [8.28, 16.69]	11.12 [8.42, 16.89]	12.57 [8.23, 16.45]	0.998
Blood Loss (mL)	1200.00 [700.00, 2000.00]	1200.00 [700.00, 2000.00]	1000.00 [700.00, 2000.00]	0.856
Crystalloids (mL)	2100.00 [1750.00, 2800.00]	2050.00 [1700.00, 2700.00]	2300.00 [1762.50, 3000.00]	0.312
Operation Duration (min)	443.00 [361.00, 520.00]	450.00 [362.75, 518.75]	415.00 [360.00, 512.50]	0.595
PRBCs (mL)	1200.00 [700.00, 1800.00]	1200.00 [800.00, 1750.00]	1200.00 [500.00, 1850.00]	0.602
Urine Output (mL)	900.00 [600.00, 1400.00]	900.00 [550.00, 1275.00]	1050.00 [700.00, 1500.00]	0.127

Values are presented as mean (SD), median [IQR], or n (%). Baseline characteristics are presented using the original, pre-imputation data

*Abbreviations* *BMI* Body Mass Index, *WBC* White Blood Cell, *Hb* Hemoglobin, *PLT* Platelet, *ALT* Alanine Aminotransferase, *AST* Aspartate Aminotransferase, *ALP* Alkaline Phosphatase, *GGT* Gamma-Glutamyl Transferase, *INR* International Normalized Ratio, *PRBCs* Packed Red Blood Cells, *MELD* Model for End-Stage Liver Disease

### Outcome definition

The primary outcome was the development of BCs within the follow-up period. BCs were defined as bile leakage, anastomotic or non-anastomotic strictures, or biliary stones, confirmed by imaging (magnetic resonance cholangiopancreatography [MRCP], endoscopic retrograde cholangiopancreatography [ERCP], or CT) or surgical exploration [20]. Outcome events were ascertained by reviewing medical records by clinicians blinded to the predictor analysis.

### Statistical analysis and sample size justification

Sample size was determined by the availability of eligible patients during the study period. In the training set, observed outcome events yielded an events per variable (EPV) ratio of 9.5, satisfying the minimum requirement (5–10) for penalized regression [21]. We further conducted a formal sample size assessment using the pmsampsize package (Riley et al.) [22]. Based on a Cox-Snell R-squared of 0.194, the calculation indicated that a minimum of 96 patients was required to minimize overfitting (Criterion 2). Our cohort of 133 patients meets this predefined requirement. To further mitigate potential overfitting given the sample size constraints, Least Absolute Shrinkage and Selection Operator (LASSO) regularization (utilizing 10-fold cross-validation) was strictly applied to constrain model complexity [23].

All statistical analyses were performed using R software (version 4.3.3). Continuous variables were compared using the Mann–Whitney U test or t-test, and categorical variables using the Chi-square or Fisher's exact test. A two-sided P-value < 0.05 was considered statistically significant.

### Model development and validation

The cohort was randomly split into a training set and a validation set at a 7:3 ratio. To strictly prevent data leakage during model training, missing data handling was performed independently after the data split. Variables with a missing rate exceeding 20% were excluded. The remaining missing values in the training and validation sets were separately imputed using Multiple Imputation by Chained Equations (MICE), assuming a missing-at-random (MAR) mechanism [24]. Continuous variables were standardized (z-score normalization) prior to model training to ensure convergence and comparability across algorithms. A staged feature selection strategy was employed within the training cohort: (1) Univariate Analysis: Univariate logistic regression was initially performed to screen candidate variables. Variables with a *P*-value < 0.1 were considered potentially relevant and advanced to the next step. (2) LASSO Regression: LASSO regression was applied to the variables identified in Step 1 to eliminate collinearity and select robust features. The optimal penalty parameter (λ) was determined via 10-fold cross-validation. (3) Multivariable Analysis & Modeling: Variables with non-zero coefficients from LASSO were entered into a multivariable logistic regression analysis. Final predictors were identified based on a combination of statistical significance and clinical relevance. Antiviral therapy was retained in the multivariable model given its clinical significance (*P* = 0.051 in univariate analysis) as a surrogate marker for hepatic inflammation. This strategy ensures the model preserves critical biological signals and enhances clinical applicability. These selected predictors were then used to train eight machine learning algorithms: Random Forest (RF), Extreme Gradient Boosting (XGBoost) [25], Light Gradient Boosting Machine (LightGBM) [26], Naïve Bayes (NB), Adaptive Boosting (AdaBoost), Support Vector Machine (SVM), K-Nearest Neighbors (KNN), and Logistic Regression (LR). Hyperparameters for ML models were optimized using 5-fold cross-validation (Additional file 1: Table S1).

Performance Evaluation: Models were evaluated in the held-out validation set using the Area Under the Curve (AUC), Brier score, and Decision Curve Analysis (DCA) [27]. Calibration curves were used to assess model calibration. The best-performing ML model was deployed as a web-based calculator (https://liver-research.shinyapps.io/Biliary-Risk-Calculator/), while the nomogram provided a visual scoring system. SHapley Additive exPlanations (SHAP) were used to interpret the ML model's predictions [14].

## Results

### Participant characteristics

**Fig. 1:**
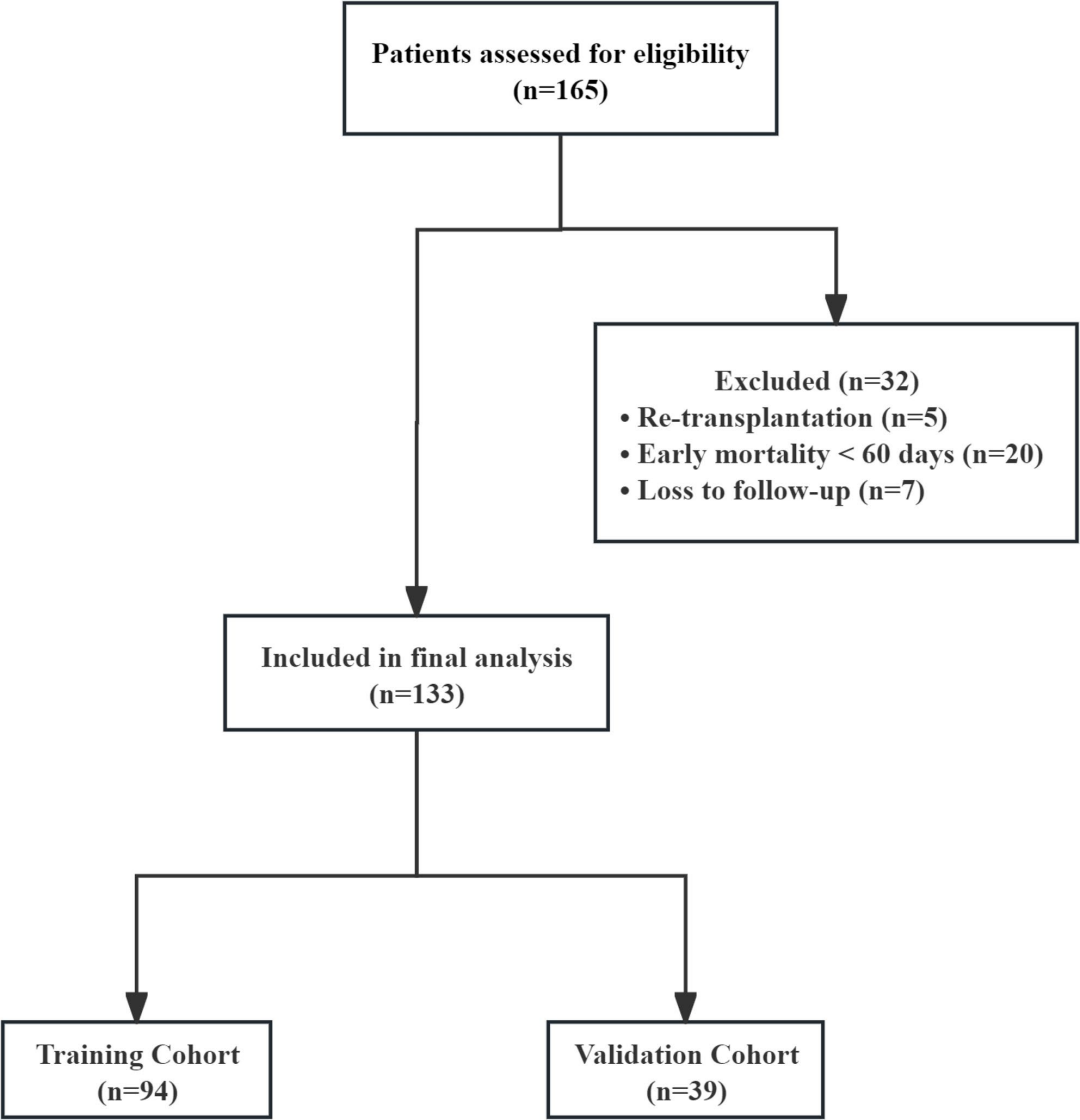
Flowchart of study participant selection. A total of 133 patients were finally enrolled and randomly divided into a training cohort (*n* = 94) and a validation cohort (*n* = 39) at a 7:3 ratio

A total of 165 patients were initially assessed for eligibility. Of these, 32 patients were excluded due to re-transplantation (*n* = 5), early mortality within 60 days (*n* = 20), or loss to follow-up (*n* = 7). Ultimately, 133 patients were included in the final analysis and were divided into a training cohort (*n* = 94) and a validation cohort (*n* = 39), as shown in the study flow diagram (Fig. 1).

The cumulative incidence of BCs was 38.3% (51/133), with a median follow-up of 38.4 months (IQR: 9.9–87.0). Regarding the transplant types, our cohort was highly homogeneous: 130 patients (97.7%) underwent deceased-donor liver transplantation (DDLT) and only 3 patients (2.3%) received living-donor liver transplantation (LDLT). Given this extreme imbalance and near-zero variance, this demographic detail was not included as an independent variable in the predictive modeling. Baseline demographic and clinical characteristics were well-balanced between the training and validation cohorts (Table 1).

Statistical analysis demonstrated that the cohorts were well-balanced, with no significant differences observed in either baseline covariates or the prevalence of the outcome (all *P* > 0.05).

### Variable screening and identification of independent predictors

**Fig. 2:**
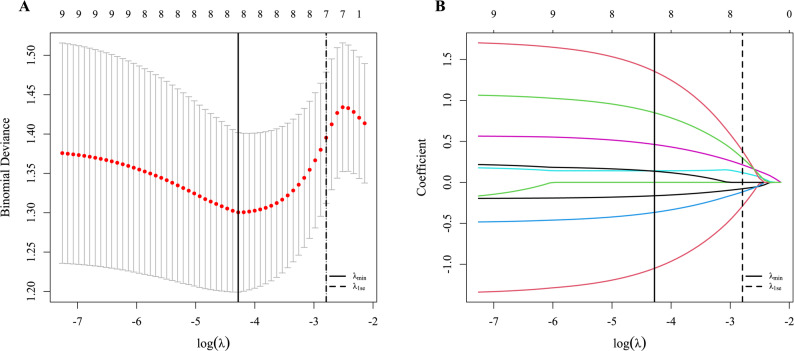
Feature selection using the LASSO logistic regression model. **A** Selection of the optimal penalization coefficient (λ) using 10-fold cross-validation. The solid vertical line marks the λ value that minimizes binomial deviance (λ_min_), while the dashed vertical line indicates the 1-standard-error rule (λ_1se_). Numbers along the top axis denote the count of predictors with non-zero coefficients at the corresponding λ. **B** LASSO coefficient profiles of the candidate clinical variables. Each curve represents the coefficient path of a specific variable against log(λ). Vertical lines indicate the optimal λ values determined in (A). Abbreviations: LASSO, least absolute shrinkage and selection operator; SE, standard error

A staged feature selection strategy was employed to identify robust predictors for BCs. Prior to formal modeling, missing data patterns were visualized (Additional file 1: Figure S1), and no variable exceeded the 20% missingness threshold. Initially, univariate logistic regression screened nine candidate variables with potential associations (*P* < 0.10). To minimize multicollinearity and isolate the most informative features, LASSO regression was applied to these candidates (Fig. 2), which refined the selection pool down to eight potential predictors.

The variables identified by LASSO were subsequently evaluated in a multivariable logistic regression model. Final predictors were determined based on a comprehensive assessment of statistical significance and clinical relevance. We retained antiviral therapy despite its borderline statistical significance (*P* = 0.051 in univariate analysis) given its established clinical relevance as a surrogate marker for hepatic inflammation.

Refitting the final model with these four predictors maximized model parsimony and clinical interpretability. Multicollinearity assessment visualized via a correlation heatmap (Additional file 1: Figure S2) confirmed that all Variance Inflation Factors (VIFs) were < 2.0 [28]. Table 2 details the parameter estimates for this final model. Specifically, HCC was identified as a significant protective factor (OR 0.185). Conversely, higher intraoperative crystalloid infusion (OR 1.916), preoperative partial hepatectomy (OR 7.550), and antiviral therapy (OR 3.104) were confirmed as independent risk factors.

**Table 2:** Multivariable logistic regression analysis for predicting biliary complications

Variable	β Coefficient	OR (95% CI)	*P* value
Hepatocellular Carcinoma	-1.689	0.185 (0.057–0.537)	0.003
Intraoperative Crystalloid Infusion (per SD increase)	0.650	1.916 (1.169–3.334)	0.014
Preoperative Partial Hepatectomy	2.022	7.550 (1.984–32.408)	0.004
Antiviral Therapy	1.133	3.104 (1.171–8.881)	0.027

For categorical variables, the odds ratio represents the risk of “Yes” compared to the reference group “No”. For continuous variables, it represents the risk per standard deviation (SD) increase. Model Constant (Intercept) = -0.6355

*CI* Confidence Interval, *OR* Odds Ratio

### Performance of machine learning models

**Fig. 3:**
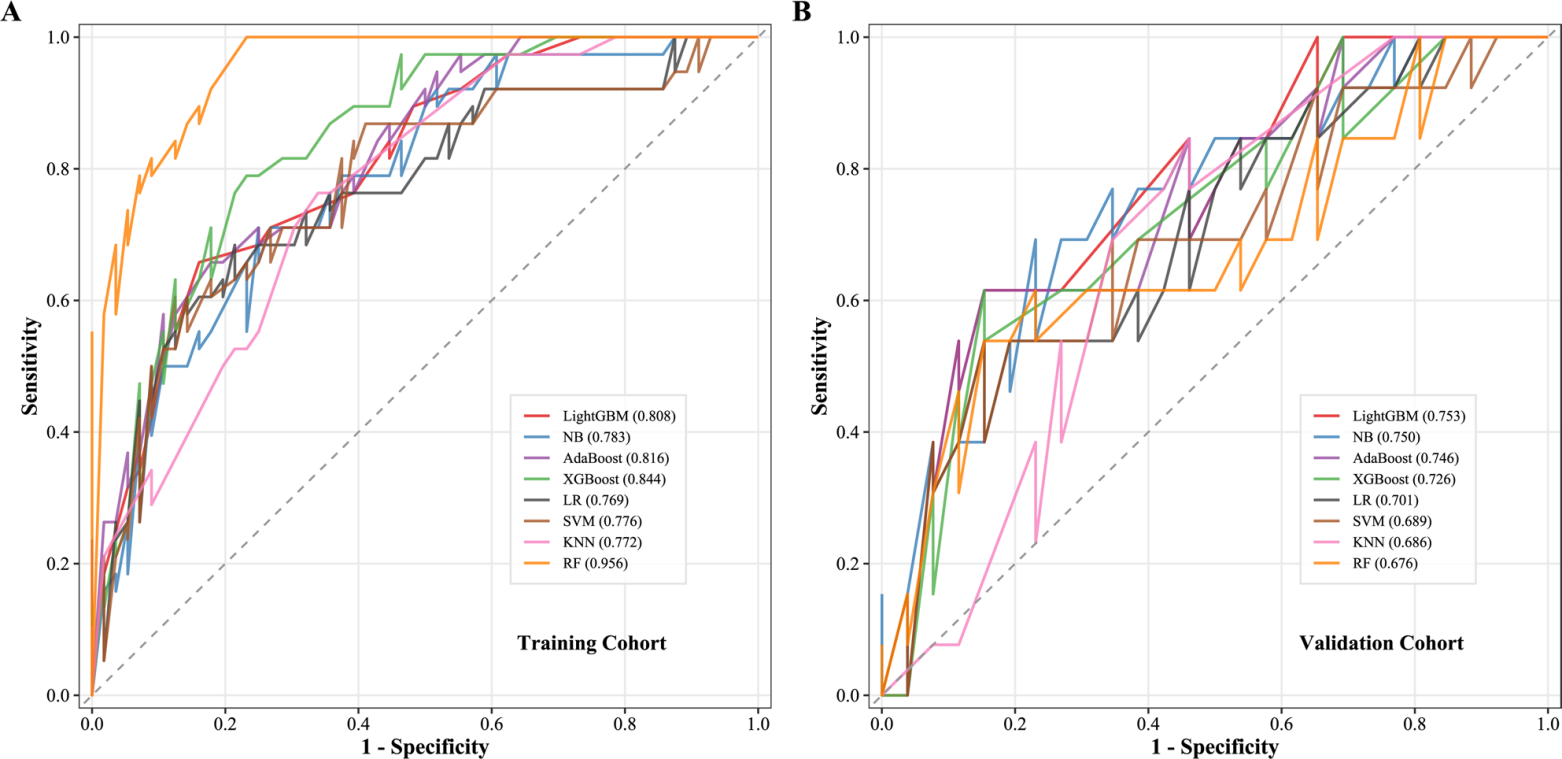
Comparative discrimination of eight machine learning models in predicting biliary complications.** A** Receiver operating characteristic (ROC) curves derived from the training cohort (*n* = 94). **B** ROC curves validated in the independent validation cohort (*n* = 39). The Random Forest model (orange curve) achieved the highest AUC in the training set (0.956) but showed a significant performance drop in the validation set (0.676), suggesting overfitting. In contrast, the LightGBM model (red curve) demonstrated the most robust generalization capability, maintaining the highest AUC (0.753) in the validation cohort. Abbreviations: ROC, receiver operating characteristic; AUC, area under the curve; LightGBM, light gradient boosting machine; XGBoost, extreme gradient boosting; AdaBoost, adaptive boosting; RF, random forest; SVM, support vector machine; NB, naïve Bayes; KNN, K-Nearest Neighbors; LR, logistic regression

The discriminative performance of eight ML algorithms was comprehensively evaluated in both the training (Fig. 3A) and independent validation cohorts (Fig. 3B).

In the validation cohort, predictive performance varied across models, as detailed in Table 3.

**Table 3:** Predictive performance of eight machine learning models in the validation cohort

Model	AUC (95% CI)	Accuracy	Sensitivity	Specificity	F1 Score	Brier Score	Optimal Threshold
LightGBM	0.753 (0.591–0.915)	0.769	0.615	0.846	0.640	0.212	0.590
NB	0.750 (0.585–0.915)	0.744	0.692	0.769	0.643	0.186	0.357
AdaBoost	0.746 (0.579–0.912)	0.769	0.615	0.846	0.640	0.198	0.491
XGBoost	0.726 (0.555–0.898)	0.769	0.615	0.846	0.640	0.207	0.636
LR	0.701 (0.524–0.879)	0.744	0.538	0.846	0.583	0.213	0.587
SVM	0.689 (0.503–0.875)	0.744	0.538	0.846	0.583	0.216	0.629
KNN	0.686 (0.521–0.852)	0.641	0.846	0.538	0.611	0.210	0.293
RF	0.676 (0.482–0.870)	0.718	0.615	0.769	0.593	0.210	0.479

The threshold represents the optimal cut-off value determined by Youden's index

*AUC* Area Under the Curve, *CI* Confidence Interval, *LightGBM* Light Gradient Boosting Machine, *NB* Naïve Bayes, *AdaBoost* Adaptive Boosting, *XGBoost* Extreme Gradient Boosting, *LR* Logistic Regression, *SVM* Support Vector Machine, *KNN* K-Nearest Neighbors, *RF* Random Forest, *F1* F1-Score

The LightGBM model demonstrated the highest discrimination, achieving an Area Under the Curve (AUC) of 0.753 (95% CI 0.591–0.915) [29]. The specific optimized hyperparameters for all evaluated machine learning models, including the final LightGBM model, are detailed in Additional file 1: Table S1. This performance was numerically superior to the standard LR model (AUC 0.701) and comparable to Naïve Bayes (AUC 0.750) and AdaBoost (AUC 0.746).

In comparison, the RF model exhibited signs of significant overfitting, with its AUC dropping markedly from 0.956 in the training set to 0.676 in the validation set. In contrast, the LightGBM model maintained stable performance, confirming its reliability on unseen data.

Beyond discrimination, the model's calibration was assessed to ensure the accuracy of predicted probabilities. The calibration plot of the LightGBM model (Additional file 1: Figure S3) visualized a high degree of agreement between the predicted and observed probabilities of BCs, supported by a Brier score of 0.212.

**Fig. 4:**
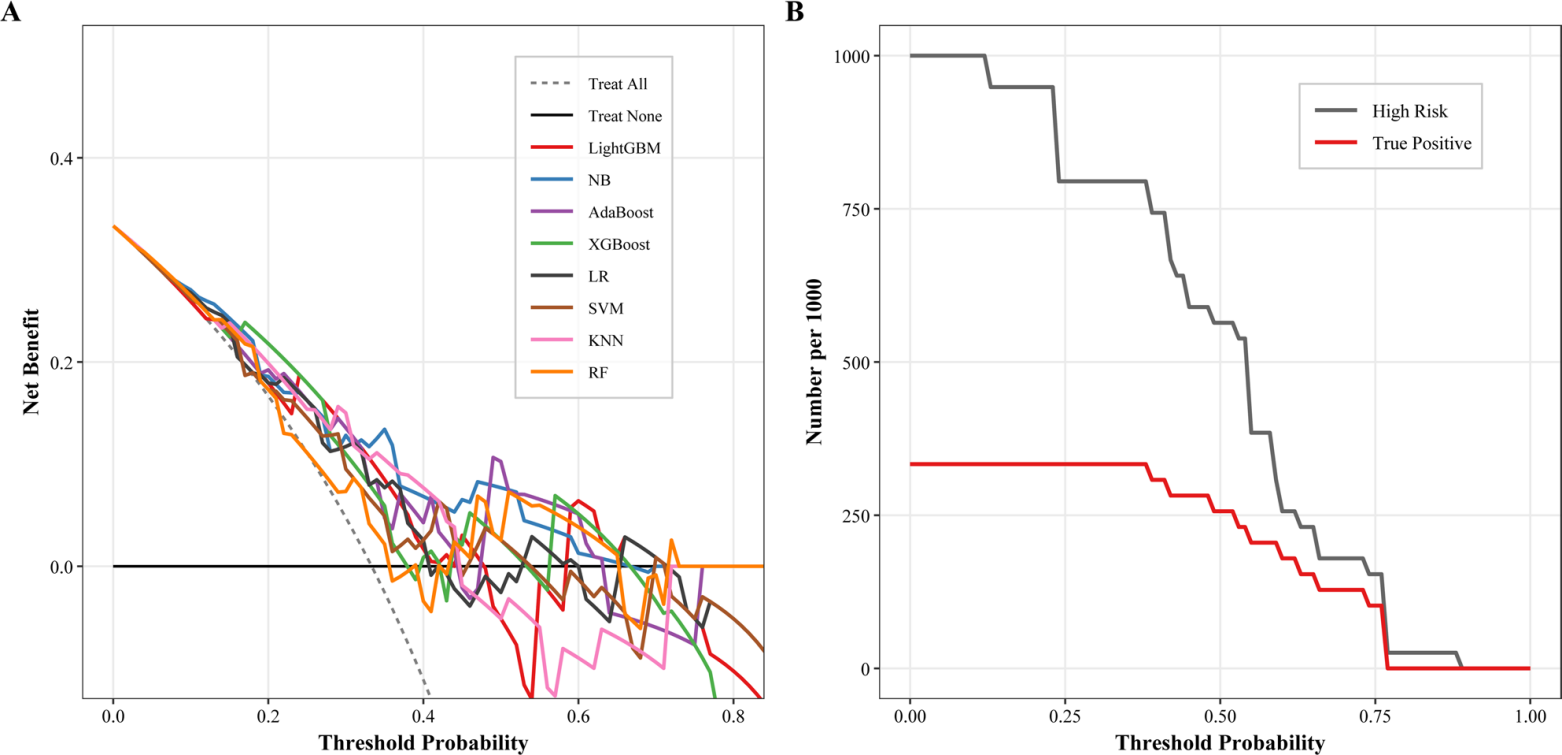
Assessment of clinical utility and diagnostic value.** A** Decision Curve Analysis (DCA) comparing the net benefit of eight machine learning models. The y-axis measures net benefit, and the x-axis represents the threshold probability. The LightGBM model (red curve) exhibited a higher net benefit compared to the “Treat All” (gray dashed line) and “Treat None” (black solid line) strategies across the clinically relevant threshold range (approximately 0.0 to 0.45). **B** Clinical Impact Curve (CIC) of the LightGBM model. The graph visualizes the estimated number of high-risk patients (gray line) versus the number of true-positive cases (red line) per 1,000 patients at varying probability thresholds. Abbreviations: DCA, decision curve analysis; CIC, clinical impact curve; LightGBM, light gradient boosting machine; NB, naïve Bayes; LR, logistic regression; RF, random forest; SVM, support vector machine; KNN, K-Nearest Neighbors; XGBoost, extreme gradient boosting; AdaBoost, adaptive boosting

To further evaluate clinical utility, Decision Curve Analysis (DCA) was performed (Fig. 4A). The results indicated that the LightGBM-based risk scoring system provided a superior net clinical benefit compared to the “treat-all” or “treat-none” strategies, particularly within the threshold probability range of 0.0 to 0.45. Although the net benefit of the ensemble model fluctuated and diminished at higher threshold probabilities (above 0.50), its robust performance in the lower threshold range (0.0 to 0.45) is especially critical for clinical screening, where high sensitivity is prioritized to preclude missing potential complications.

Furthermore, the Clinical Impact Curve (Fig. 4B) corroborated these findings [30]. It visually demonstrated that while a discrepancy between predicted high-risk patients and actual cases existed at lower thresholds, the two curves began to converge significantly at threshold probabilities exceeding 0.60. This convergence indicates that at higher specificity levels, the LightGBM model possesses excellent diagnostic accuracy with a minimal false-positive rate, thereby reinforcing its practical value in risk stratification and clinical decision-making.

### Model interpretability and variable importance

**Fig. 5:**
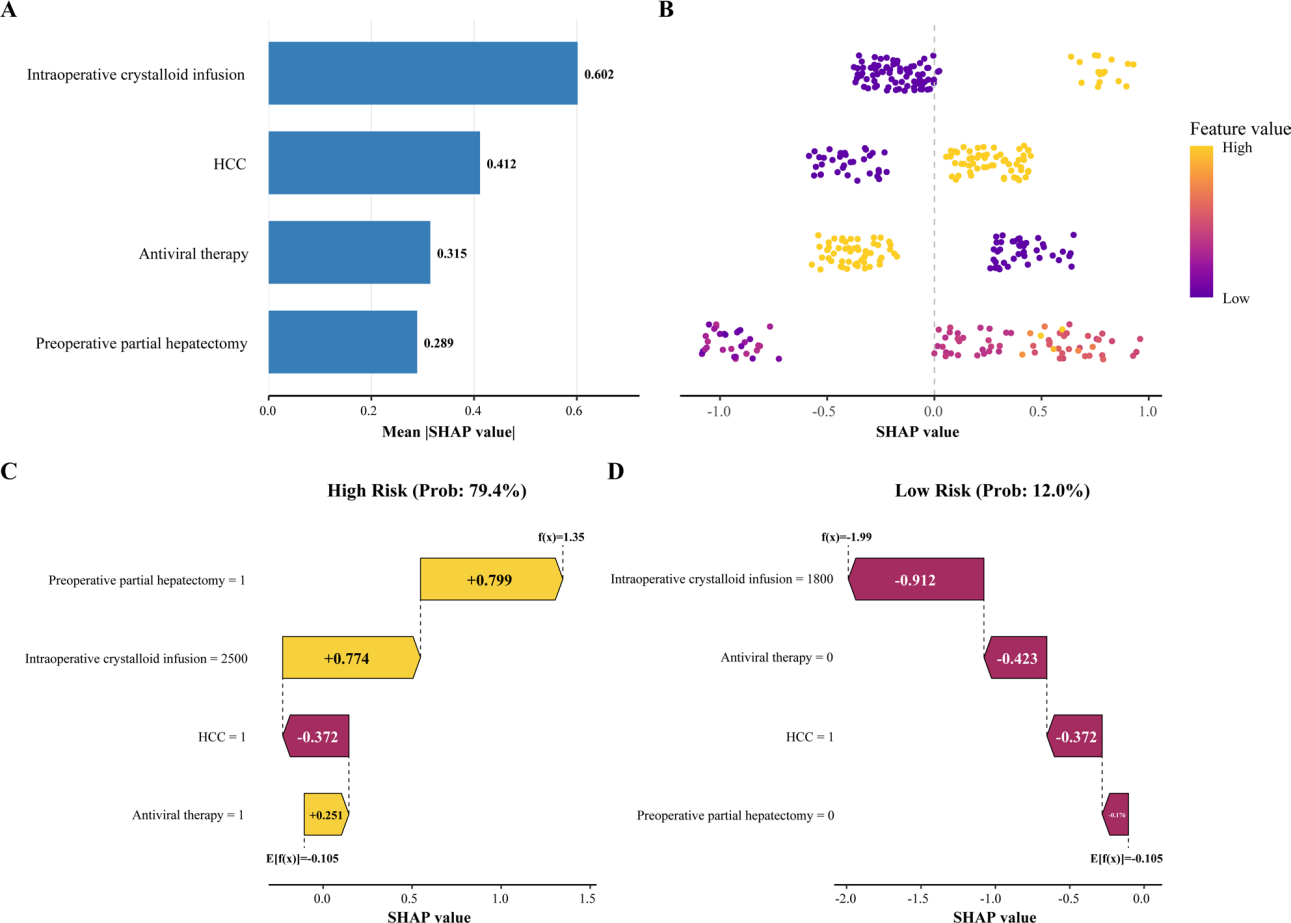
SHAP analysis for model interpretability.** A** Global feature importance ranking based on mean absolute SHAP values. Intraoperative crystalloid infusion was identified as the top predictor. **B** SHAP beeswarm plot illustrating feature effects. Each dot represents a patient, with color indicating feature value (yellow: high; purple: low). Higher crystalloid infusion volumes (yellow dots) correlate with positive SHAP values, indicating increased risk. **C**,** D** Waterfall plots showing decision paths for representative patients. Yellow bars indicate risk-increasing factors; purple bars indicate risk-decreasing factors. **C** A high-risk case (probability: 79.4%), where preoperative partial hepatectomy and high intraoperative crystalloid infusion (2,500 mL) were the primary drivers of the high predicted risk. **D** A low-risk case (probability: 12.0%), where lower crystalloid volume (1,800 mL) and absence of antiviral therapy significantly reduced the risk score. Abbreviations: SHAP, SHapley Additive exPlanations; HCC, hepatocellular carcinoma; LightGBM, light gradient boosting machine

To enhance the interpretability of the LightGBM model and visualize the specific contribution of each predictor, we employed SHapley Additive exPlanations (SHAP) analysis. The global feature importance ranking is presented in Fig. 5A. Intraoperative crystalloid infusion was identified as the most influential predictor, followed by HCC, antiviral therapy, and preoperative partial hepatectomy. The beeswarm plot (Fig. 5B) further illustrates the directionality of these effects. Specifically, higher volumes of crystalloid infusion (represented by yellow dots) were strongly associated with positive SHAP values, indicating an increased risk of BCs.

To illustrate the model's utility in personalized risk assessment, we visualized the decision path for representative individual patients (Fig. 5C and D). In a high-risk case (Predicted Probability: 79.4%), preoperative partial hepatectomy and high intraoperative crystalloid infusion (2,500 mL) were the primary drivers of the elevated score (Fig. 5C). In contrast, a patient with a moderate crystalloid volume (1,800 mL) and absence of antiviral therapy was classified as low risk (Predicted Probability: 12.0%), despite the presence of other baseline risk factors (Fig. 5D).

**Fig. 6:**
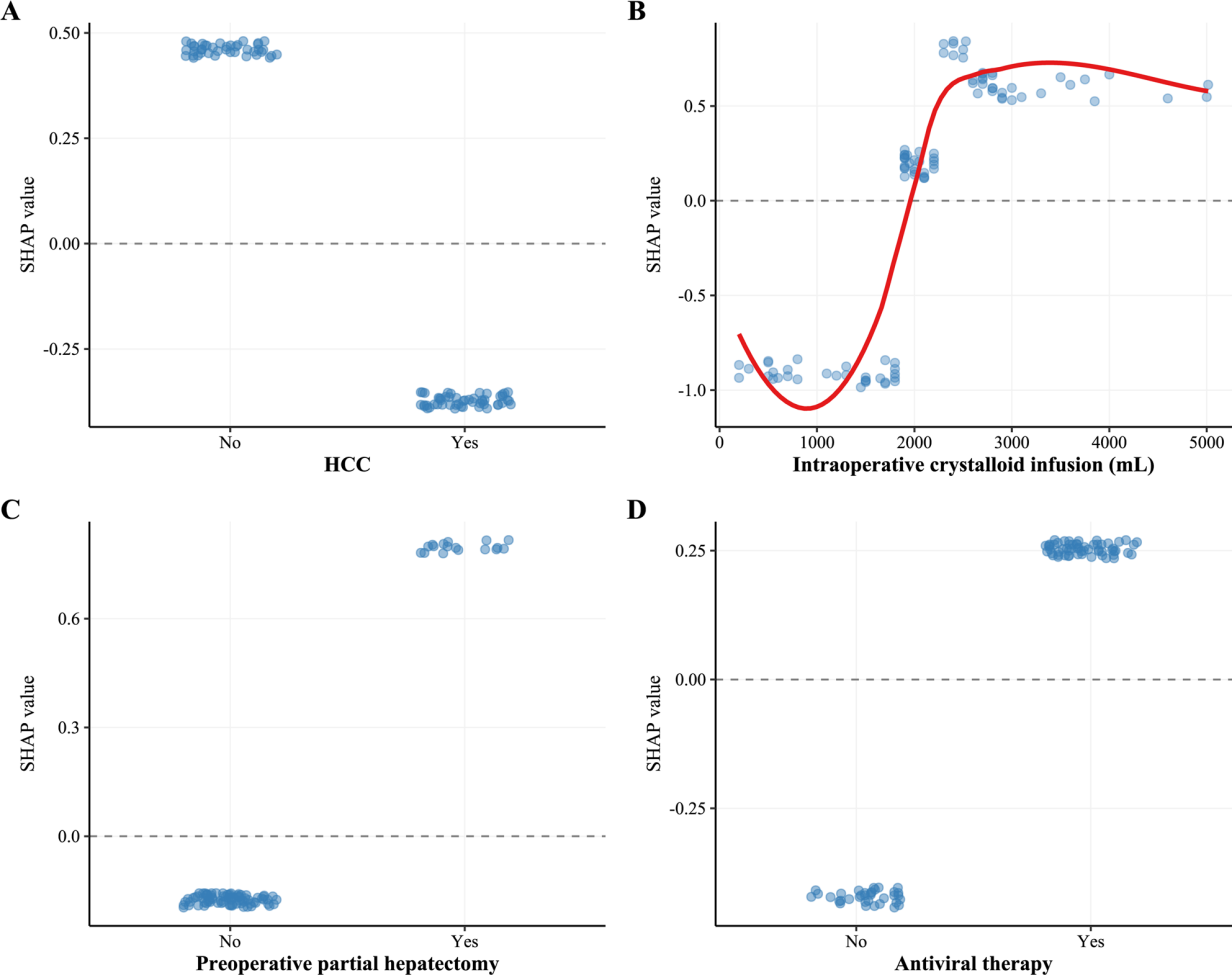
SHAP dependence plots illustrating non-linear relationships between key predictors and biliary complication risk. SHAP values > 0 indicate a positive contribution to risk, while values < 0 indicate a protective effect. **A** Hepatocellular Carcinoma (HCC). The presence of HCC (Yes) is associated with negative SHAP values, reflecting a relatively lower risk compared to non-HCC indications. **B** Intraoperative Crystalloid Infusion. A distinct non-linear threshold effect is observed; risk escalates sharply as infusion volume exceeds ~ 1,500 mL and plateaus after 2,000 mL, crossing the zero-threshold at approximately 1,800 mL. **C** Preoperative Partial Hepatectomy. A history of partial hepatectomy (Yes) consistently contributes positively to the predicted risk. **D** Antiviral Therapy. Patients requiring antiviral therapy (Yes) show elevated SHAP values, indicating increased susceptibility. Abbreviations: SHAP, SHapley Additive exPlanations; HCC, hepatocellular carcinoma

Finally, to capture the non-linear relationships between clinical features and outcomes, SHAP dependence plots were generated (Fig. 6). A clear threshold effect was observed for intraoperative crystalloid infusion (Fig. 6B): the risk of BCs escalated sharply when the infusion volume exceeded approximately 1,500–2,000 mL, eventually reaching a plateau. The presence of HCC (Fig. 6A) was associated with negative SHAP values, suggesting a relatively lower risk compared to other indications in this study cohort. Conversely, both preoperative partial hepatectomy (Fig. 6C) and antiviral therapy (Fig. 6D) demonstrated a positive contribution to the predicted risk.

### Development of a clinical prediction tool

**Fig. 7:**
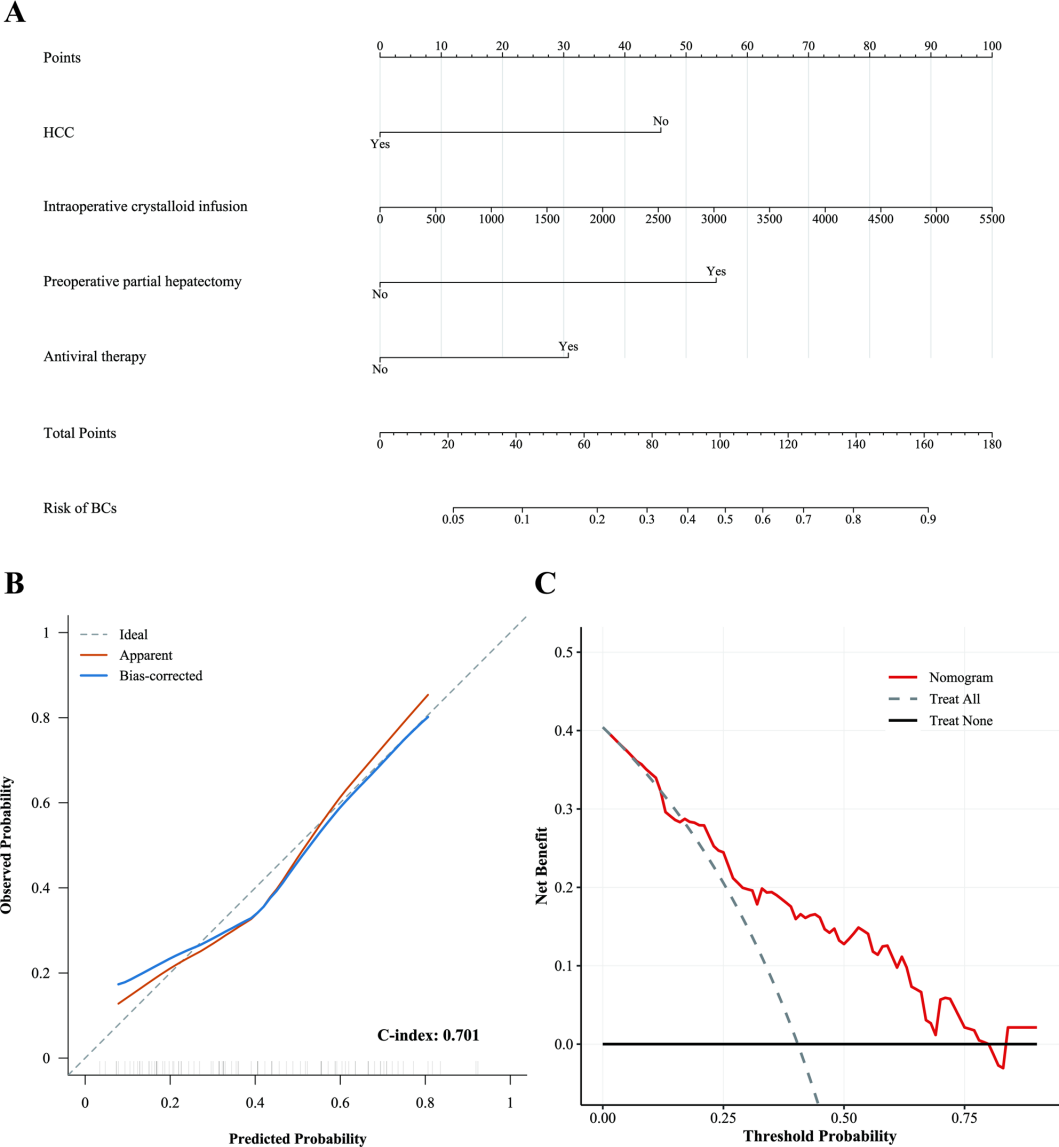
Construction and validation of a nomogram for predicting biliary complications.** A** Quantitative nomogram integrating four independent predictors. To estimate an individual's risk, locate the value for each variable on its respective axis, draw a vertical line upward to the “Points” scale, and sum the points. The total score is projected onto the bottom “Risk of BCs” scale. Intraoperative crystalloid infusion spans the widest point range (0–100), reflecting its dominant predictive weight. Consistent with SHAP analysis, the presence of HCC is assigned 0 points, while its absence contributes approximately 46 points, aligning with its role as a protective factor relative to non-HCC indications. **B** Calibration curve assessed in the validation cohort. The bias-corrected curve (blue solid line) closely tracks the ideal diagonal (grey dashed line), indicating excellent agreement between predicted and observed probabilities (C-index: 0.701). **C** Decision Curve Analysis (DCA). The red line represents the nomogram's net benefit. The analysis demonstrates that using the nomogram to guide intervention provides superior net benefit compared to “treat-all” or “treat-none” strategies across a wide range of threshold probabilities (0.05 to 0.85). Abbreviations: HCC, hepatocellular carcinoma; DCA, decision curve analysis; BCs, biliary complications

To translate the identified risk factors into an accessible clinical instrument, we constructed a quantitative nomogram (Fig. 7A). This graphical tool integrates the four key predictors—HCC status, intraoperative crystalloid infusion, preoperative partial hepatectomy, and antiviral therapy—allowing clinicians to estimate the individual probability of BCs by summing the points assigned to each variable. Consistent with the multivariate analysis, intraoperative crystalloid infusion contributes the broadest range of points to the total score (0 to approximately 100 points), reflecting its strong predictive weight. Consistent with SHAP analysis, the presence of HCC is assigned 0 points, whereas its absence contributes approximately 46 points.

The performance of this nomogram was rigorously validated. The bias-corrected calibration curve (Fig. 7B) demonstrated good agreement between the nomogram-predicted probabilities and the actual observed frequencies, with a concordance index (C-index) of 0.701. Decision Curve Analysis (Fig. 7C) confirmed the clinical utility of the nomogram, providing a positive net benefit across a broad threshold range of 0.05 to 0.85. This represents a wider spectrum of clinical applicability compared to the LightGBM model, which exhibited its optimal net benefit within the 0.0 to 0.45 range.

Risk Stratification. Based on the Youden index (threshold 0.44, score 95), patients were stratified into high- and low-risk groups. In the training set, the high-risk group exhibited a significantly higher BC incidence (68.4% vs. 21.4%, *P* < 0.001). When applied to the independent validation cohort, the model maintained a similar discriminatory trend. Patients stratified as high-risk in the validation set exhibited a notably higher rate of BCs compared to the low-risk group (44.4% vs. 23.8%). Although this absolute risk difference (20.6%) did not reach statistical significance (*P* = 0.196)—likely due to the limited validation sample size—the trend aligned with the training cohort.

While the nomogram provides a convenient static visualization, ensemble machine learning models offer superior precision in capturing non-linear interactions. Therefore, to leverage the optimal discriminative power of the LightGBM algorithm, we developed a dynamic, web-based risk calculator. This digital interface allows clinicians to input the four variables and instantly obtain a personalized risk probability for BCs. The online tool is freely accessible via the internet (https://liver-research.shinyapps.io/Biliary-Risk-Calculator/) [31]. This combined framework—providing both a bedside nomogram for quick visual reference and a web-based calculator for precision—ensures the model's applicability across diverse clinical settings.

## Discussion

In this study, we developed and validated an exploratory machine learning and nomogram framework for biliary complications (BCs) after liver transplantation (LT) using real-world clinical data. The cumulative incidence of BCs observed in our cohort was 38.3%, which is slightly higher than some literature estimates (10–30%). This is primarily attributed to our comprehensive definition of BCs—encompassing not only major strictures but also early minor leakages and late-onset stones—and our relatively long median follow-up of 38.4 months, allowing for the capture of late-onset complications. By integrating a staged feature selection strategy with machine learning (ML) algorithms, we identified four robust predictors: hepatocellular carcinoma (HCC), intraoperative crystalloid infusion, preoperative partial hepatectomy, and antiviral therapy. Our findings demonstrate that the LightGBM model outperformed standard LR and other ML algorithms, offering superior discrimination (AUC 0.753) and calibration. To enhance clinical utility, we deployed this model as both a high-precision web-based calculator and a visually interpretable nomogram, balancing algorithmic precision with clinical usability.

### Interpretation of key predictors

The four identified predictors reflect the interaction between baseline liver status, surgical history, and intraoperative management.

- *HCC (Confounding Protective Factor)*: HCC was identified as a protective factor against BCs. Rather than a true biological protective effect conferred by the malignancy, this finding predominantly reflects selection bias and confounding by the comparatively preserved hepatic reserve in these recipients. Quantitative analysis of our cohort confirmed that HCC recipients possessed significantly better physiological reserves compared to non-HCC recipients, characterized by lower MELD scores (median 9.83 vs. 14.70, P < 0.001), higher albumin levels (median 39.00 vs. 34.30 g/L, P < 0.001), and superior coagulation function (median INR 1.17 vs. 1.56, P < 0.001). These patients typically present with compensated cirrhosis, contrasting with the decompensated state (e.g., massive ascites, liver failure) frequently observed in benign end-stage liver disease. This preserved metabolic state provides a favorable biological foundation for postoperative tissue repair and anastomotic healing [32, 33].
- *Preoperative Partial Hepatectomy*: A history of partial hepatectomy emerged as the strongest independent risk factor in our cohort (OR 7.550). Prior hepatobiliary surgery typically induces severe hilar adhesions and anatomical distortion, which substantially elevate the technical difficulty of biliary reconstruction during transplantation [34]. Furthermore, previous surgical dissection may disrupt the delicate peribiliary vascular plexus, thereby compromising arterial perfusion to the bile duct remnant and rendering the anastomosis susceptible to ischemic complications [35].
- *Intraoperative Crystalloid Infusion*: The data demonstrated a significant volume-dependent association between crystalloid infusion volume and the risk of BCs (OR 1.916 per SD increase). Excessive crystalloid administration promotes fluid extravasation, inevitably leading to visceral and graft edema. This pathological edema elevates interstitial pressure, which mechanically compresses the microvasculature and impairs perfusion to the biliary epithelium—a tissue notably vulnerable to ischemic insult—thereby compromising anastomotic healing, a pathophysiological cascade well-documented in major abdominal surgery and highly applicable to the biliary tract [36, 37].
- *Antiviral Therapy*: Although initially borderline significant during variable selection, antiviral therapy was confirmed as a significant independent predictor in the final multivariate model (P = 0.027). This variable likely functions solely as a potential surrogate marker for a persistent pro-inflammatory microenvironment rather than a direct causative agent. Patients requiring antiviral therapy typically possess a history of active viral replication and chronic hepatic inflammation.

### Model performance and comparison

Unlike previous studies that primarily focused on donor-related risk factors, our model specifically targets recipient and intraoperative variables. While traditional scoring systems like MELD focus primarily on waitlist mortality, they lack specificity for predicting postoperative surgical complications. Our study highlights the incremental value of ML techniques in this niche. The LightGBM model demonstrated superior discrimination compared to conventional LR (AUC 0.753 vs. 0.701) and exhibited superior calibration.

This performance advantage likely stems from the gradient boosting algorithm's inherent ability to capture non-linear relationships and high-order interactions—such as the threshold effect of crystalloid infusion visualized in our SHAP plots—which standard linear models may not fully capture [38]. Beyond discriminatory metrics, our DCA provides a clinical rationale for our combined framework. While the LightGBM-based system functions as a high-precision engine for high-sensitivity screening at lower risk thresholds, the nomogram offers a more resilient and stable framework that maintains positive net benefit across a significantly broader probability spectrum (0.05 to 0.85).

Consequently, although the traditional nomogram showed reduced discriminatory power, it remains indispensable for practical bedside application. By integrating the advanced LightGBM web calculator for precision estimation with the pragmatic nomogram for rapid screening, we successfully balance algorithmic complexity with clinical usability [39], ensuring the model's adaptability across diverse clinical settings.

### Clinical implications

Our findings support shifting from reactive management to proactive risk stratification.

- *Preoperative Stratification*: Clinicians should regard patients with a history of partial hepatectomy or active viral status as "high-risk" candidates. For these individuals, surgical planning should anticipate difficult hilar dissection, and comprehensive preoperative counseling regarding biliary risks is warranted [34].
- *Intraoperative Optimization*: The strong link between crystalloid overload and BCs aligns with current Enhanced Recovery After Surgery (ERAS) principles. We advocate for a goal-directed fluid therapy (GDFT) strategy, incorporating fluid-restrictive principles to minimize visceral edema, provided hemodynamic stability is meticulously maintained [37].
- *Postoperative Surveillance*: For high-risk patients identified by the web calculator, routine observation may be insufficient. We recommend an intensified surveillance protocol, such as lowering the threshold for early MRCP, enabling timely intervention before irreversible biliary failure occurs [18].

### Limitations

#### This study has several limitations

First, the sample size (*n* = 133), while sufficient for initial model development, remains relatively modest for high-dimensional ML algorithms. Although we employed rigorous cross-validation and LASSO regularization, the modest overall sample size and the low number of outcome events (*n* = 13) in the validation cohort introduce a substantial risk of overfitting and instability in model estimates [22]. This statistical uncertainty is reflected in the wide 95% confidence intervals for the AUC, limiting the robustness of the reported performance metrics [16, 22]. Consequently, this study must be interpreted strictly as an exploratory, proof-of-concept framework and an internal validation effort, rather than a clinically implementable prediction tool.

Second, the retrospective, single-center design introduces inherent selection bias and limits the generalizability of our findings. The model's performance may be partially influenced by institution-specific surgical techniques and perioperative management protocols. Therefore, our results serve as a foundational model requiring corroboration in diverse practice settings.

Third, we acknowledge the critical absence of detailed transplant-specific and donor-specific variables (e.g., precise cold/warm ischemia times, graft steatosis, and graft-to-recipient weight ratio). Because our historical database spans 14 years (2011–2025), our early recipient electronic medical record (EMR) system was inherently isolated from the separate organ procurement network. Consequently, these granular donor parameters were not systematically documented in the recipients' clinical charts and could not be reliably retrieved retrospectively. Although our cohort is highly homogeneous (97.7% DDLT), this omission limits the model's comprehensive predictive capacity.

Finally, although internal validation demonstrated robust discrimination and calibration, external validation in larger, multi-center cohorts is imperative to confirm the model's transportability and clinical utility before widespread adoption, in accordance with the latest TRIPOD + AI 2024 reporting standards [16].

## Conclusions

In conclusion, this proof-of-concept study developed and internally validated an exploratory machine learning and nomogram framework for post-transplant BCs, integrating a high-precision LightGBM-based web calculator with a clinically interpretable nomogram. By integrating four independent predictors (HCC, preoperative partial hepatectomy, intraoperative crystalloid infusion, and antiviral therapy), our model captures the interaction between recipient baseline characteristics and intraoperative management. The LightGBM algorithm demonstrated superior discrimination and calibration compared to conventional LR, highlighting the value of ML in addressing non-linear clinical interactions. This strategy enables clinicians to transition from reactive management to proactive risk stratification, facilitating individualized perioperative optimization (e.g., GDFT) and targeted postoperative surveillance. Future efforts should focus on external validation in large-scale, multi-center cohorts to confirm the model's generalizability and support its integration into routine clinical practice. 

## Supplementary Information

Supplementary Material 1: This file contains Figure S1 (Visualization of missing data patterns in the study cohort); Figure S2 (Correlation heatmap for multicollinearity assessment); Figure S3 (Calibration plot of the LightGBM model in the validation cohort); and Table S1 (Hyperparameter optimization details for the evaluated machine learning models).

## Data Availability

The datasets generated and/or analysed during the current study are available from the corresponding author on reasonable request.

## Abbreviations

AdaBoost: Adaptive Boosting
ALP: Alkaline Phosphatase
ALT: Alanine Aminotransferase
AST: Aspartate Aminotransferase
AUC: Area Under the Curve
BCs: Biliary Complications
BMI: Body Mass Index
CI: Confidence Interval
CIC: Clinical Impact Curve
CT: Computed Tomography
DCA: Decision Curve Analysis
DDLT: Deceased-Donor Liver Transplantation
EMR: Electronic Medical Record
ERAS: Enhanced Recovery After Surgery
ERCP: Endoscopic Retrograde Cholangiopancreatography
F1: F1-Score
GDFT: Goal-Directed Fluid Therapy
GGT: Gamma-Glutamyl Transferase
Hb: Hemoglobin
HBV: Hepatitis B Virus
HCC: Hepatocellular Carcinoma
INR: International Normalized Ratio
IQR: Interquartile Range
KNN: K-Nearest Neighbors
LASSO: Least Absolute Shrinkage and Selection Operator
LDLT: Living-Donor Liver Transplantation
LightGBM: Light Gradient Boosting Machine
LR: Logistic Regression
LT: Liver Transplantation
MELD: Model for End-Stage Liver Disease
MICE: Multiple Imputation by Chained Equations
ML: Machine Learning
MRCP: Magnetic Resonance Cholangiopancreatography
MRI: Magnetic Resonance Imaging
NB: Naïve Bayes
OR: Odds Ratio
PLT: Platelet
PRBCs: Packed Red Blood Cells
RF: Random Forest
ROC: Receiver Operating Characteristic
SD: Standard Deviation
SHAP: SHapley Additive exPlanations
SVM: Support Vector Machine
VIF: Variance Inflation Factor
WBC: White Blood Cell
XGBoost: Extreme Gradient Boosting
