# Supplementary material for: Predicting biliary complications after liver transplantation: a machine learning and nomogram-based exploratory study
Source: BMC Gastroenterol. 2026 May 12;26:408. doi: 10.1186/s12876-026-04924-0 (PMC13401290; doi:10.1186/s12876-026-04924-0)
Supplement: Supplementary file 1 — Supplementary Material 1: This file contains Figure S1 (Visualization of missing data patterns in the study cohort); Figure S2 (Correlation heatmap for multicollinearity assessment); Figure S3 (Calibration plot of the LightGBM model in the validation cohort); and Table S1 (Hyperparameter optimization details for the evaluated machine learning models). [file 12876_2026_4924_MOESM1_ESM.docx]

**Additional file 1**

**Title:** Supplementary figures and table for: Predicting Biliary Complications After Liver Transplantation: A Machine Learning and Nomogram-Based Exploratory Study

Han Zhang¹, Jinxi Chen¹, Minghang Zhang¹, Kezhen Zong¹*, Shanshan Li¹*, Zuotian Huang²*, Zhongjun Wu¹*

¹ Liver Transplantation Center, The First Affiliated Hospital of Chongqing Medical University, No. 1 Youyi Road, Yuzhong District, Chongqing 400016, China.

² Department of Hepatobiliary Pancreatic Tumor Center, Chongqing University Cancer Hospital, No. 181, Hanyu Road, Shapingba District, Chongqing 400030, China.

***Corresponding author:** Kezhen Zong (zongkezhen23@163.com); Shanshan Li (3520473684@qq.com); Zuotian Huang (1351619201@qq.com); Zhongjun Wu (wzjtcy@126.com)

**Table of Contents**

**Table S1.** Hyperparameter optimization details for the evaluated machine learning models Page 3

**Supplementary Figures**

**Figure S1.** Visualization of missing data patterns in the study cohort Page 6

**Figure S2.** Correlation heatmap for multicollinearity assessment Page 8

**Figure S3.** Calibration plot of the LightGBM model in the validation cohort Page 10

**Table S1.** Hyperparameter optimization details for the evaluated machine learning models.

| **Model** | **Hyperparameter** | **Description** | **Search Space** | **Selected Value** |
| --- | --- | --- | --- | --- |
| LightGBM (Best Model) | num_leaves | Maximum tree leaves for base learners | {2, 3, 4} | 2 |
|  | learning_rate | Shrinkage rate applied to each step | {0.01, 0.05} | 0.05 |
|  | min_data_in_leaf | Minimum number of records in a leaf | {10, 15, 20} | 10 |
|  | lambda_l1 | L1 regularization term on weights | {0.1, 0.5} | 0.5 |
|  | lambda_l2 | L2 regularization term on weights | {0.1, 0.5} | 0.1 |
| NB | fL | Laplace correction smoothing parameter | {0, 0.5, 1} | 0 |
|  | adjust | Bandwidth adjustment for density estimation | {1, 1.5} | 1.5 |
|  | usekernel | Use kernel density estimate | TRUE (Fixed) | TRUE |
| AdaBoost | mfinal | Number of iterations / trees | {50, 100, 150} | 150 |
|  | maxdepth | Maximum depth of individual trees | {1, 2} | 1 |
| XGBoost | nrounds | Number of boosting iterations | 1–100 (CV Optimized) | 96 |
|  | max_depth | Maximum depth of a tree | 2 (Fixed) | 2 |
|  | eta | Learning rate | 0.1 (Fixed) | 0.1 |
|  | subsample | Subsample ratio of the training instances | 0.8 (Fixed) | 0.8 |
| RF | mtry | Number of variables randomly sampled | {1, 2, 3, 4} | 2 |
|  | splitrule | Splitting rule for node division | {gini, extratrees} | gini |
|  | min.node.size | Minimum size of terminal nodes | {1, 3, 5} | 3 |
|  | ntree | Number of trees grown | 1000 (Fixed) | 1000 |
| SVM | sigma | Inverse width parameter for the RBF kernel | {0.01, 0.05, 0.1, 0.2} | 0.01 |
|  | C | Cost of constraints violation | {0.1, 0.5, 1, 2, 5} | 5 |
| KNN | k | Number of nearest neighbors | 1–19 (step 2) | 7 |
| LR | - | Standard Maximum Likelihood Estimation | N/A | N/A |

**Note:** Hyperparameters were optimized using 5-fold cross-validation grid search to maximize model performance while rigorously preventing overfitting. Values marked as "Fixed" were predetermined based on established best practices for the sample size. **Abbreviations:** AdaBoost, adaptive boosting; CV, cross-validation; KNN, k-nearest neighbors; LightGBM, light gradient boosting machine; LR, logistic regression; NB, naïve Bayes; RBF, radial basis function; RF, random forest; SVM, support vector machine; XGBoost, extreme gradient boosting.


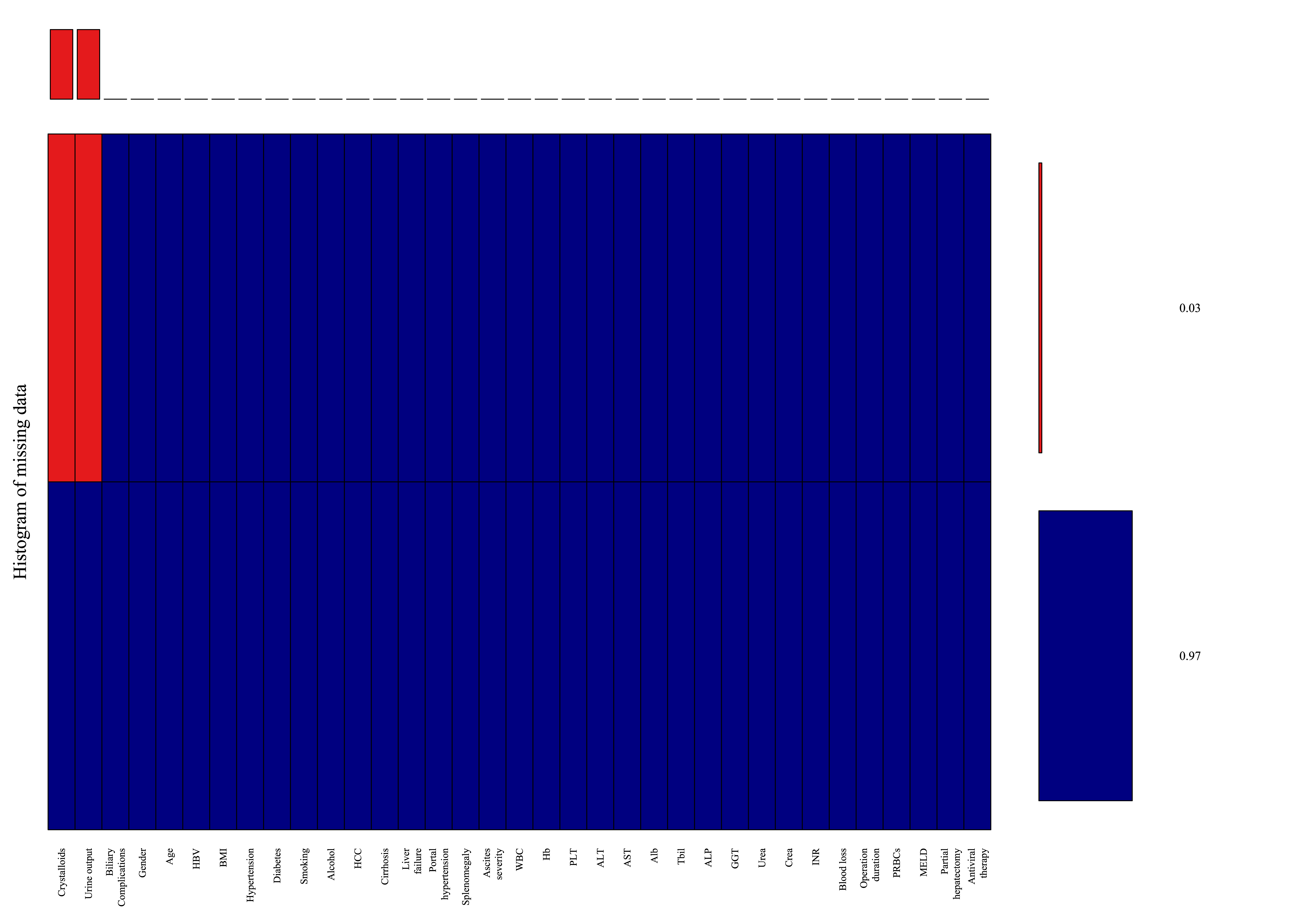


**Figure S1.** Visualization of missing data patterns in the study cohort.

**Legend:** Missing data map for all candidate variables before imputation. The x-axis represents the clinical variables, and the y-axis represents the patient samples. Blue areas indicate observed data, while red areas indicate missing values. All variables included in the subsequent analysis had a missing rate of less than 20%, satisfying the criteria for Multiple Imputation by Chained Equations (MICE). Note: Certain variable names have been abbreviated for formatting purposes. **Abbreviations:** Alb, Albumin; ALP, Alkaline Phosphatase; ALT, Alanine Aminotransferase; AST, Aspartate Aminotransferase; BMI, Body Mass Index; Blood loss, Intraoperative blood loss; Crea, Creatinine; Crystalloids, Intraoperative crystalloid infusion; GGT, Gamma-Glutamyl Transferase; Hb, Hemoglobin; HBV, Hepatitis B Virus; HCC, Hepatocellular Carcinoma; INR, International Normalized Ratio; MELD, Model for End-Stage Liver Disease; Partial hepatectomy, Preoperative partial hepatectomy; PLT, Platelet; PRBCs, Packed Red Blood Cells; Tbil, Total Bilirubin; WBC, White Blood Cell


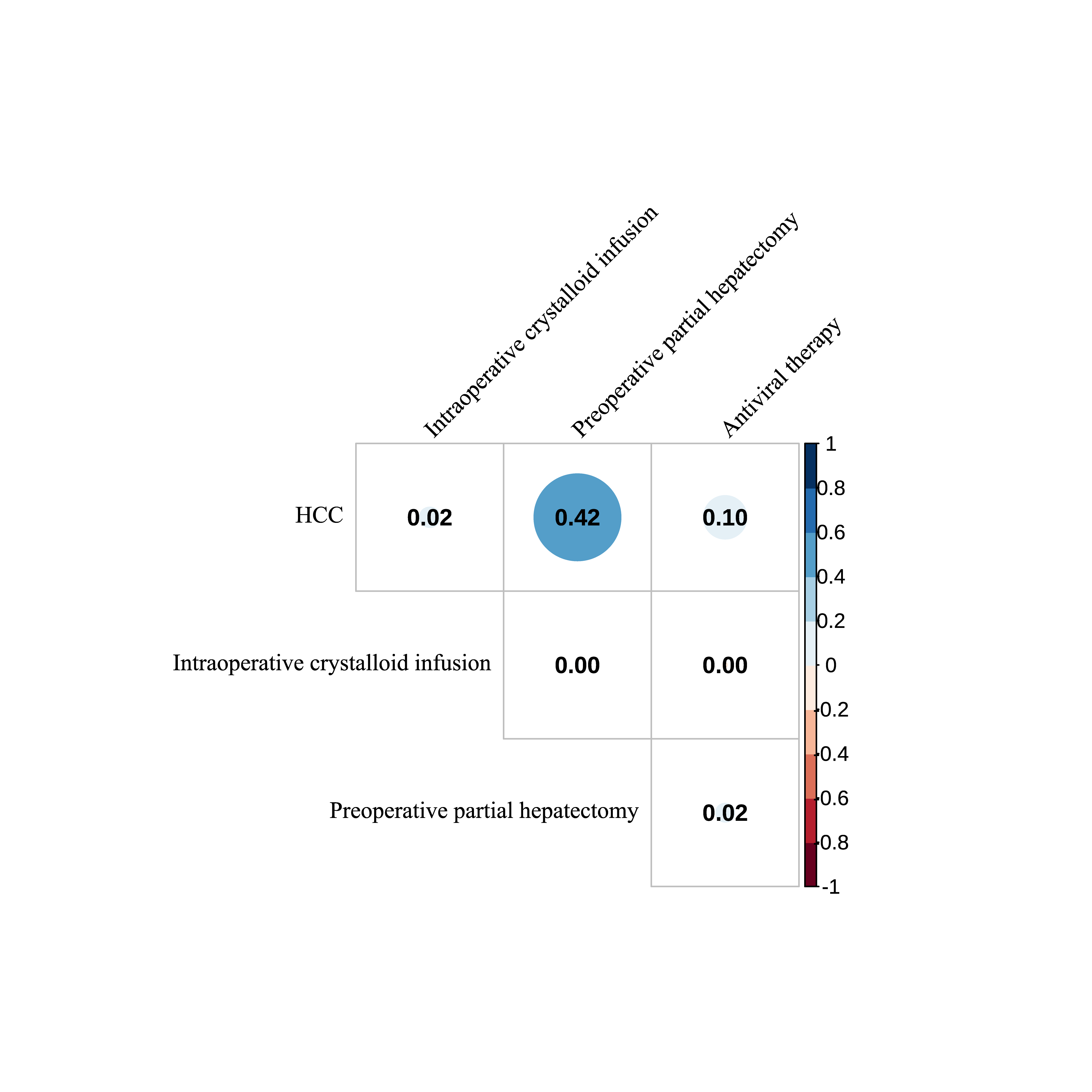
**Figure S2.** Correlation heatmap for multicollinearity assessment.

**Legend:** Heatmap of the correlation matrix among the final candidate predictors identified by LASSO regression. The color gradient, ranging from dark red (-1.0) to dark blue (1.0), represents the correlation coefficients between variables. The highest observed correlation was moderate (r = 0.42) between HCC and preoperative partial hepatectomy, with no strong correlations (typically defined as |r| > 0.7) observed among any of the selected variables. Subsequent Variance Inflation Factor (VIF) analysis confirmed that all VIF values were strictly < 2.0, definitively ruling out significant multicollinearity. **Abbreviations:** HCC, Hepatocellular Carcinoma.


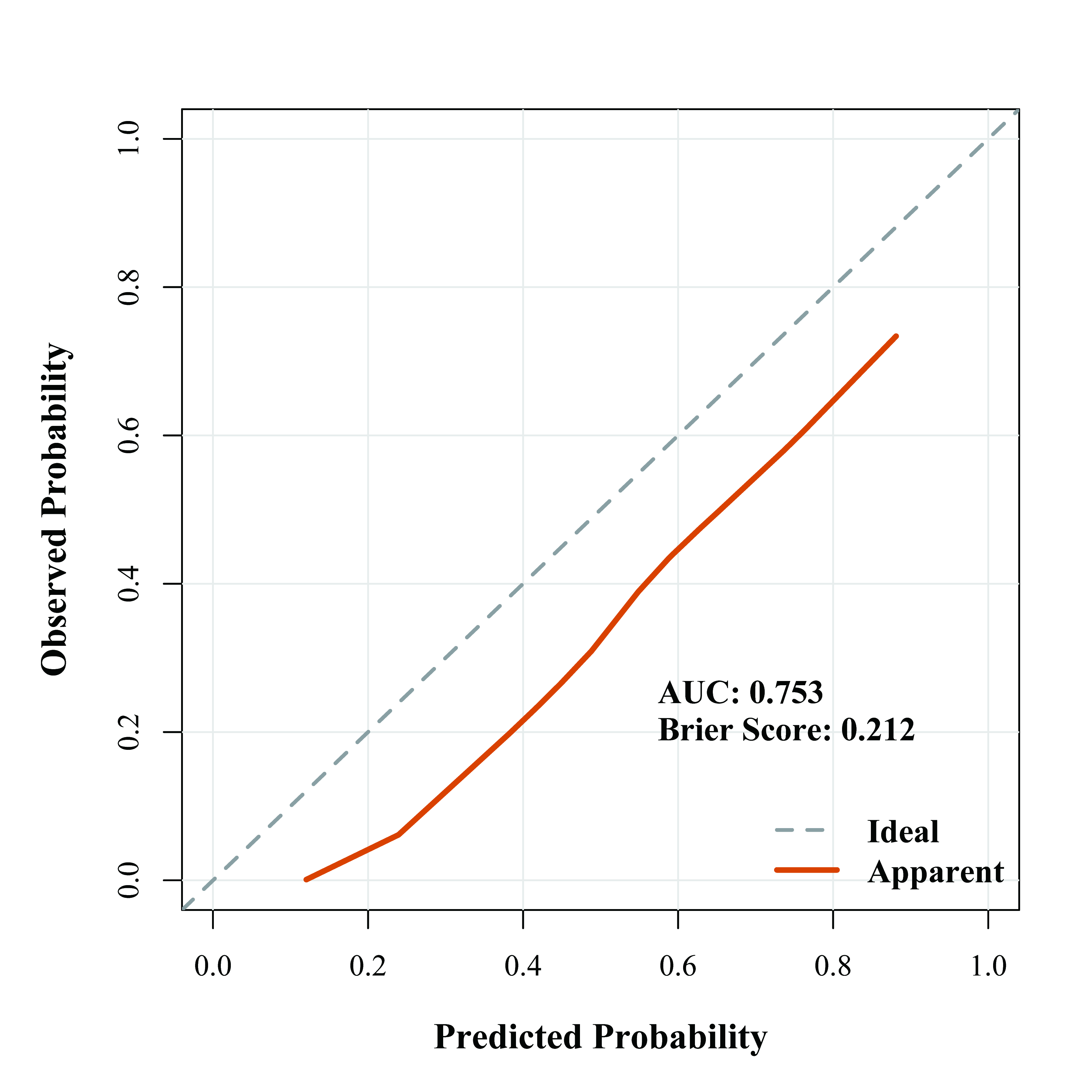


**Figure S3.** Calibration plot of the LightGBM model in the validation cohort.

**Legend:** The bias-corrected calibration curve assesses the agreement between the LightGBM-predicted probability of biliary complications (x-axis) and the actual observed frequency (y-axis) in the independent validation set. The grey dashed line represents perfect calibration (ideal scenario). The orange solid line represents the apparent calibration performance of the model. The model exhibited satisfactory calibration with an AUC of 0.753 and a Brier score of 0.212. **Abbreviations:** AUC, Area Under the Curve; LightGBM, Light Gradient Boosting Machine.
